# Supplementary material for: Modeling glioblastoma heterogeneity as a dynamic network of cell states
Source: Mol Syst Biol. 2021 Sep 16;17(9):e10105. doi: 10.15252/msb.202010105 (PMC8444284; doi:10.15252/msb.202010105)
Supplement: Supplementary file 5 — Source Data for Figure 3 [file MSB-17-e10105-s001.zip › Figure3A_sourcedata/GSEA_3065/hallmarks_state5.GseaPreranked.1623416302988/HALLMARK_COMPLEMENT.html]

Details for gene set HALLMARK\_COMPLEMENT[GSEA]

|  || Dataset | state5 |
| Phenotype | NoPhenotypeAvailable |
| Upregulated in class | na\_pos |
| GeneSet | HALLMARK\_COMPLEMENT |
| Enrichment Score (ES) | 0.32555312 |
| Normalized Enrichment Score (NES) | 1.3048652 |
| Nominal p-value | 0.057142857 |
| FDR q-value | 0.16263716 |
| FWER p-Value | 0.513 |
Table: GSEA Results Summary

  

Fig 1: Enrichment plot: HALLMARK\_COMPLEMENT      
 Profile of the Running ES Score & Positions of GeneSet Members on the Rank Ordered List

  

| PROBE | GENE SYMBOL | GENE\_TITLE | RANK IN GENE LIST | RANK METRIC SCORE | RUNNING ES | CORE ENRICHMENT || 1 | PLAT |  |  | 37 | 0.714 | 0.0293 | Yes |
| 2 | HSPA5 |  |  | 43 | 0.698 | 0.0615 | Yes |
| 3 | LAMP2 |  |  | 69 | 0.584 | 0.0861 | Yes |
| 4 | COL4A2 |  |  | 93 | 0.540 | 0.1089 | Yes |
| 5 | PRCP |  |  | 103 | 0.523 | 0.1324 | Yes |
| 6 | CD46 |  |  | 104 | 0.523 | 0.1569 | Yes |
| 7 | TIMP1 |  |  | 109 | 0.518 | 0.1807 | Yes |
| 8 | TFPI2 |  |  | 124 | 0.504 | 0.2028 | Yes |
| 9 | CLU |  |  | 162 | 0.474 | 0.2209 | Yes |
| 10 | CTSV |  |  | 193 | 0.443 | 0.2383 | Yes |
| 11 | LRP1 |  |  | 259 | 0.404 | 0.2500 | Yes |
| 12 | CTSC |  |  | 347 | 0.362 | 0.2572 | Yes |
| 13 | CD59 |  |  | 354 | 0.360 | 0.2734 | Yes |
| 14 | CTSD |  |  | 388 | 0.347 | 0.2860 | Yes |
| 15 | ADAM9 |  |  | 421 | 0.334 | 0.2981 | Yes |
| 16 | CPM |  |  | 439 | 0.328 | 0.3115 | Yes |
| 17 | TIMP2 |  |  | 451 | 0.325 | 0.3256 | Yes |
| 18 | DOCK10 |  |  | 751 | 0.250 | 0.3037 | No |
| 19 | CTSB |  |  | 902 | 0.225 | 0.2974 | No |
| 20 | PSEN1 |  |  | 1217 | 0.183 | 0.2706 | No |
| 21 | SERPINE1 |  |  | 1221 | 0.182 | 0.2789 | No |
| 22 | RAF1 |  |  | 1275 | 0.175 | 0.2811 | No |
| 23 | FN1 |  |  | 1342 | 0.167 | 0.2815 | No |
| 24 | CTSL |  |  | 1384 | 0.163 | 0.2846 | No |
| 25 | C1R |  |  | 1625 | 0.140 | 0.2641 | No |
| 26 | L3MBTL4 |  |  | 1695 | 0.134 | 0.2627 | No |
| 27 | USP15 |  |  | 1717 | 0.132 | 0.2665 | No |
| 28 | MMP15 |  |  | 1942 | 0.116 | 0.2467 | No |
| 29 | LGMN |  |  | 2091 | 0.106 | 0.2351 | No |
| 30 | AKAP10 |  |  | 2328 | 0.090 | 0.2128 | No |
| 31 | LIPA |  |  | 2451 | 0.082 | 0.2029 | No |
| 32 | GNAI3 |  |  | 2504 | 0.079 | 0.2008 | No |
| 33 | RCE1 |  |  | 2748 | 0.065 | 0.1765 | No |
| 34 | PLAUR |  |  | 2826 | 0.061 | 0.1707 | No |
| 35 | IRF1 |  |  | 2926 | 0.056 | 0.1622 | No |
| 36 | USP8 |  |  | 3184 | 0.042 | 0.1353 | No |
| 37 | CPQ |  |  | 3428 | 0.031 | 0.1094 | No |
| 38 | GPD2 |  |  | 3594 | 0.024 | 0.0919 | No |
| 39 | CTSO |  |  | 3624 | 0.023 | 0.0897 | No |
| 40 | VCPIP1 |  |  | 3987 | 0.008 | 0.0494 | No |
| 41 | LTA4H |  |  | 4159 | 0.000 | 0.0302 | No |
| 42 | RNF4 |  |  | 4272 | -0.005 | 0.0178 | No |
| 43 | PCLO |  |  | 4551 | -0.016 | -0.0127 | No |
| 44 | DYRK2 |  |  | 4718 | -0.023 | -0.0303 | No |
| 45 | SIRT6 |  |  | 4765 | -0.025 | -0.0343 | No |
| 46 | USP14 |  |  | 4831 | -0.027 | -0.0404 | No |
| 47 | PLSCR1 |  |  | 5043 | -0.034 | -0.0625 | No |
| 48 | CA2 |  |  | 5139 | -0.038 | -0.0714 | No |
| 49 | SH2B3 |  |  | 5209 | -0.041 | -0.0772 | No |
| 50 | USP16 |  |  | 5327 | -0.045 | -0.0882 | No |
| 51 | CASP4 |  |  | 5377 | -0.048 | -0.0915 | No |
| 52 | GCA |  |  | 5488 | -0.052 | -0.1014 | No |
| 53 | IRF7 |  |  | 5524 | -0.054 | -0.1028 | No |
| 54 | PRDM4 |  |  | 5609 | -0.058 | -0.1096 | No |
| 55 | XPNPEP1 |  |  | 5652 | -0.060 | -0.1115 | No |
| 56 | GMFB |  |  | 5812 | -0.066 | -0.1263 | No |
| 57 | CASP7 |  |  | 5942 | -0.072 | -0.1374 | No |
| 58 | IRF2 |  |  | 6031 | -0.076 | -0.1438 | No |
| 59 | PIK3CA |  |  | 6045 | -0.077 | -0.1416 | No |
| 60 | PREP |  |  | 6101 | -0.080 | -0.1441 | No |
| 61 | RBSN |  |  | 6610 | -0.109 | -0.1961 | No |
| 62 | EHD1 |  |  | 6613 | -0.109 | -0.1912 | No |
| 63 | STX4 |  |  | 6687 | -0.113 | -0.1941 | No |
| 64 | CSRP1 |  |  | 6689 | -0.113 | -0.1889 | No |
| 65 | FYN |  |  | 6823 | -0.123 | -0.1981 | No |
| 66 | GNG2 |  |  | 6841 | -0.124 | -0.1942 | No |
| 67 | ZEB1 |  |  | 6944 | -0.132 | -0.1995 | No |
| 68 | RABIF |  |  | 6945 | -0.132 | -0.1933 | No |
| 69 | KIF2A |  |  | 7030 | -0.139 | -0.1962 | No |
| 70 | CASP3 |  |  | 7082 | -0.144 | -0.1952 | No |
| 71 | CEBPB |  |  | 7185 | -0.154 | -0.1994 | No |
| 72 | GNB4 |  |  | 7251 | -0.162 | -0.1991 | No |
| 73 | DUSP6 |  |  | 7258 | -0.162 | -0.1922 | No |
| 74 | MSRB1 |  |  | 7458 | -0.187 | -0.2058 | No |
| 75 | GRB2 |  |  | 7531 | -0.195 | -0.2047 | No |
| 76 | RHOG |  |  | 7585 | -0.202 | -0.2012 | No |
| 77 | PDP1 |  |  | 7777 | -0.229 | -0.2120 | No |
| 78 | FDX1 |  |  | 7788 | -0.232 | -0.2022 | No |
| 79 | PSMB9 |  |  | 7929 | -0.262 | -0.2057 | No |
| 80 | LAP3 |  |  | 8062 | -0.288 | -0.2070 | No |
| 81 | PPP4C |  |  | 8225 | -0.332 | -0.2096 | No |
| 82 | MT3 |  |  | 8279 | -0.348 | -0.1992 | No |
| 83 | PPP2CB |  |  | 8347 | -0.369 | -0.1895 | No |
| 84 | CALM3 |  |  | 8517 | -0.443 | -0.1877 | No |
| 85 | GNAI2 |  |  | 8540 | -0.452 | -0.1690 | No |
| 86 | LGALS3 |  |  | 8550 | -0.460 | -0.1484 | No |
| 87 | ANXA5 |  |  | 8626 | -0.510 | -0.1329 | No |
| 88 | CALM1 |  |  | 8748 | -0.609 | -0.1180 | No |
| 89 | GNB2 |  |  | 8790 | -0.644 | -0.0924 | No |
| 90 | S100A13 |  |  | 8820 | -0.670 | -0.0642 | No |
| 91 | ATOX1 |  |  | 8851 | -0.712 | -0.0342 | No |
| 92 | PFN1 |  |  | 8977 | -1.045 | 0.0008 | No |
Table: GSEA details [plain text format]

  

Fig 2: HALLMARK\_COMPLEMENT: Random ES distribution      
 Gene set null distribution of ES for **HALLMARK\_COMPLEMENT**
